# Low Risk Perception about Ticks and Tick-Borne Diseases in an Area Recently Invaded by Ticks in Northwestern Italy

**DOI:** 10.3390/vetsci8070131

**Published:** 2021-07-13

**Authors:** Aitor Garcia-Vozmediano, Giorgia Giglio, Elisa Ramassa, Fabrizio Nobili, Luca Rossi, Laura Tomassone

**Affiliations:** 1 Department of Veterinary Sciences, University of Turin, L.go Braccini, 2, 10095 Grugliasco, Italy; aitor.garciavozmediano@unito.it (A.G.-V.); giorgia.giglio@edu.unito.it (G.G.); luca.rossi@unito.it (L.R.); 2 Ente di Gestione delle Aree Protette delle Alpi Cozie, Via Fransuà Fontan, 1, 10050 Salbertrand, Italy; ramassa@alpicozie.eu; 3 Ente di Gestione delle Aree Protette del Po Torinese, Corso Trieste 98, 10024 Moncalieri, Italy; fabrizio.nobili@collinatorinese.org

**Keywords:** *Ixodes ricinus*, tick-borne pathogens, risk perception, Alpine area, Italy

## Abstract

Risk perception, together with the adoption of measures to prevent tick bites, may strongly influence human exposure to ticks and transmitted pathogens. We created a questionnaire to evaluate how people perceive the health risk posed by ticks in an area recently invaded by these arthropods, in the western Italian Alps. Moreover, through a collaborative effort with park rangers and physicians, we investigated which tick species bite humans and their infection with pathogens (*Borrelia burgdorferi* s.l., *Anaplasma phagocytophilum,* and spotted-fever group Rickettsiae). Apart from two *Dermacentor marginatus* bites, we identified *Ixodes ricinus* (*n* = 124) as the main species responsible for tick bites. The investigated pathogens infected 25.4% of tested *I. ricinus*. The evaluation of the engorgement rate of biting *I. ricinus* revealed that they had been likely feeding on humans for 24 h or more, suggesting a high probability of pathogen transmission. Indeed, the questionnaires revealed that people infrequently adopt preventive measures, such as inspecting the body for ticks, although most respondents claimed that ticks are a threat to human health. Having suffered from previous tick bites was positively associated with the adoption of personal protection measures. Given the increasing incidence of tick-borne diseases in the region, the public should be better informed about the possibility of being bitten by infected ticks in order to mitigate the risk.

## 1. Introduction

Lyme borreliosis and tick-borne encephalitis (TBE), transmitted by *Ixodes ricinus*, are the most common tick-borne diseases reported in Europe and their incidence has been increasing over the past decades [1,2]. In parallel, *I. ricinus,* the most abundant tick vector in Europe [3], has expanded its geographical distribution into areas at higher altitude or latitude, up until now deemed unsuitable [4]. Optimal habitats for *I. ricinus* include moist and shady woodlands, leaf litter, and medium to large-sized wildlife; consequently, these habitats represent risk areas for tick bites and for contracting tick-borne diseases. *Dermacentor* spp. are also expanding their distribution range in Europe, and are emerging as disease vectors [5]; they can transmit several viruses (e.g., TBEV) and Rickettsiales [3]. *Dermacentor* are open country tick species, also occurring in urban and suburban areas [5]. It was reported that they bite humans, although at lower rates than *I. ricinus*. 

The risk of exposure to infected ticks increases when time is spent outdoors for recreational or occupational purposes [6]. However, the perceived risk of tick bites and tick-borne infections, together with the adoption of preventive and protective measures, may strongly influence human exposure to ticks and transmitted pathogens [7,8,9]. This perceived risk might be particularly low in areas that have been recently invaded by ticks, where people may not be aware of the possibility of being bitten and are not used to adopt preventive measures. 

In our study area, located in the northwestern Italian Alps, ticks have expanded their geographic range, and recently invaded mountain zones at higher altitudes than previously recorded. This phenomenon may be linked to several factors, such as changes in land-use practices and spontaneous reforestation, increased abundance of wild ungulate populations, and the milder temperatures that mountain areas are experiencing due to climatic changes. In Piedmont, ticks were deemed rare until the early 2000s, while they now can be found on vegetation, up to approximately 1700 m above sea level [10,11]. *Ixodes ricinus* is the prevalent tick species, and it can harbor different zoonotic bacteria, including *Borrelia burgdorferi* s.l. [10]. Notifications of Lyme borreliosis in humans have increased in recent years, with the Piedmont Regional Service for the Epidemiology of Infectious Diseases (SEREMI) reporting some 20 cases per year in 2018 and 2019 (around 0.46 cases/100,000 inhabitants), compared to only eight cases reported in the period 1990 to 2008 [12]. A similar increase in reported cases was documented in the neighboring Lombardy region, which experienced a maximum of 0.26 new cases per 100,000 inhabitants in 2014 [13]. By contrast, higher rates have been recorded in the Swiss Alpine Cantons of Valais and Ticino, with 50 to 100 cases/100,000 reported in the period 2008–2011 [14]. *Dermacentor marginatus* ticks are also present in Piedmont, in cohabitation with *D. reticulatus* in hilly areas, and their infection with spotted fever group (SFG) rickettsiae was demonstrated [11]. 

Due to the novelty of the tick-related health threat in Piedmont, we investigated ticks biting humans and their infections by tick-borne bacteria. Moreover, through a questionnaire, we aimed to evaluate the level of risk awareness in the population, and the attitudes of respondents in relation to ticks and the diseases they transmit. 

## 2. Materials and Methods

### 2.1. Collection and Analysis of Ticks Feeding on Humans

The study was carried out in Turin province, Piedmont region, northwestern Italy. From spring 2017 to autumn 2019, we conducted a passive surveillance activity by targeting ticks feeding on forest rangers and visitors of the Alpi Cozie and the Po Torinese protected areas. The first is a regional Alpine park, while Po Torinese is a periurban hilly natural park located nearby Turin city; details on the study areas can be found in [11]. Tick collection was done under the framework of a scientific collaboration between the parks and the University of Turin, aimed at studying tick spread and phenology in both natural areas. In 2018–2019, we also collected ticks from human patients visited by general physicians in Susa Valley and/or presented at the emergency room of Susa Hospital. Ticks were removed either by the subjects themselves or by the physician on duty; patients were asked to sign informed consent and data were anonymously treated. Results of laboratory testing were returned to collaborating physicians or the park personnel.

Collected ticks were individually preserved in 70% ethanol and morphologically identified to stage and species level by using a stereomicroscope and identification keys [3,15]. To estimate the attachment duration of ticks on humans, we first determined the tick engorgement index (TEI-Index 2) [16], expressed as the ratio between the total body length and the maximum *scutum* width. Then, we related the TEI index with the tick attachment duration for *I. ricinus* nymphs and females through non-linear regression equations [17].

When possible, we collected the main epidemiological data associated with tick bite events, including gender and age of the bitten person, the estimated date, the geographic location of the tick bite, and the activity carried out when the bite occurred. Regarding the ticks collected by physicians, we developed an online questionnaire, asking the health status of the patients, any laboratory tests, and the treatment, when applicable.

### 2.2. Laboratory Analyses

We performed DNA extraction from *I. ricinus* using the DNAzol reagent^®^ (Life Technologies LTD, Warrington, United Kingdom), as previously described [10]. Eight *I. ricinus* specimens were not analyzed because they were damaged or badly conserved. Ticks were subjected to conventional PCR targeting the intergenic spacer region 5S and 23S rRNA of *Borrelia burgdorferi* s.l. [18], and a fragment of *gltA* [19] and *OmpA* genes [20] of *Rickettsia* spp. We also looked for *Anaplasma phagocytophilum* through qPCR (*msp2* gene) [21] and positive samples were further subjected to an end-point *groEL* gene PCR [22]. Positive and water negative controls were used in all molecular assays performed.

Obtained amplicons were purified using the ExoSAP-IT™ PCR Product Clean-up Kit (GE Healthcare Limited, Chalfont, UK) and sent to an external service (BMR Genomics, Padua, Italy) for automatic sequencing.

### 2.3. Tick Awareness Evaluation

Together with the personnel of Alpi Cozie and Po Torinese natural parks, we carried out several informative meetings during the tick seasons (spring–summer), 2017 to 2019. The meetings were addressed to the general public and to specific community categories particularly exposed to tick bites, namely hunters, mushroom pickers, and hikers. During these meetings, we mainly described our findings on the increased range expansion of ticks on the territory; we discussed the ecology of ticks and transmitted pathogens, and provided recommendations to prevent tick bites and, thus, reduce the risk of contracting tick-borne diseases. At the beginning of the meetings, we distributed a simple questionnaire in Italian among the participants, who were asked to respond anonymously. The questionnaire included closed and open questions and was aimed at evaluating the awareness level about ticks and associated health hazards (Supplementary Materials Figure S1). Personal information regarding age, gender, and employment of respondents were additionally requested. 

### 2.4. Statistical Analyses

Prevalence of infection by pathogens in ticks was calculated, with 95% exact binomial confidence intervals (CI). The Fisher’s exact test was used to study differences among categorical variables. Logistic regression was applied to model the association of age (divided in four classes: 1: ≤30 years, 2: 30–50, 3: 51–70, 4: ≥71), gender, category of respondents (general public, hikers, mushroom pickers, hunters), and previous experiences of tick bites, with the probability of adopting preventive measures against tick bites; odds ratios (OR) were calculated with 95%CI. Analyses were performed with RStudio [23]; *p*-values lower than *p* = 0.05 were considered statistically significant.

## 3. Results

### 3.1. Collection of Ticks Feeding on Humans

From 2017 to 2020, we collected 126 Ixodid ticks from 119 bitten people, including forest rangers (*n* = 33), park visitors (*n* = 58) and patients who turned to physicians for medical care (*n* = 28). *Ixodes ricinus* was the main species responsible for tick bites. We identified 8 larvae, 98 nymphs, and 16 adults; the life stage of two specimens was not determined because ticks had been damaged during removal. Nymphs were involved in 76 tick bite events, followed by adult females (*n* = 15), larvae (*n* = 6), and adult males (*n* = 1). Immature stages co-occurred only in two cases, including one larva and a single nymph feeding on a young boy scout, and one larva together with eight nymphs collected from an adult nature guide. Moreover, two *Dermacentor marginatus* adults (one male, one female) were collected from two different patients referred to the emergency unit of Susa Hospital.

We calculated the tick engorgement index (TEI) by measuring 108 ticks, whose bodies were intact after the removal. *Ixodes ricinus* TEI values averaged 2.1 (minimum–maximum = 1.6–2.6) in larvae, 2.3 (minimum–maximum = 1.5–5) in nymphs, and 2.6 (minimum–maximum = 1.8–4.5) in females. The male and the female of *D. marginatus* recorded TEI-values of 1.4 and 4.8, respectively. 

The duration of tick attachment, estimated for *I. ricinus* in nymphs (*n* = 86) and females (*n* = 13), always exceeded 24 h. Most of the ticks were removed within the first 72 h after the tick bite, although in some cases this time probably overtook 96 h (Table 1).

We recorded tick bite events in Alpine and hilly locations. They especially occurred during spring, with 42.5% of the events registered in June; 36.3% of bites occurred from March to May and 21.2% from July to October. The two *D. marginatus* bites occurred in April and May. Most tick bites (*n* = 98) could be associated with outdoor activities; of these, 61.2% (95% CI = 50.8–70.9) occurred during leisure activities, including sport training, walking and mushroom picking, while 38.8% (95% CI = 29.1–49.1) were associated with working activities (e.g., forestry rangers, camping staff). Unfortunately, we had no information on the specific activity carried out by 21 people when the bite occurred. 

**Figure 1:**
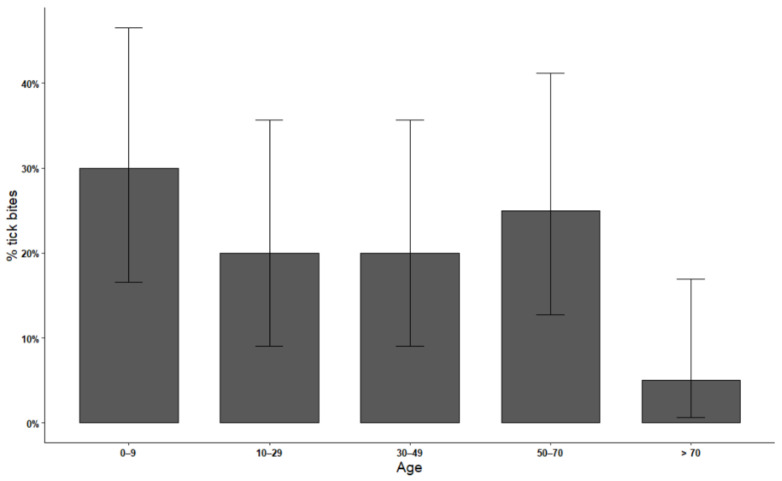
Prevalence of tick bites recorded in 2018–2019 by collaborating physicians, with 95% CI, according to age categories (in years).

Data on the age of patients were available for the 39 tick bite events registered by collaborating physicians in 2018–2019. One-third of bites occurred in young age categories (Fisher’s exact test, *p* < 0.01; Figure 1), in particular children. Eighteen bites were recorded in the category 40–69 years of age, and only two cases were reported in elderly people, over 70 years old. Five patients experienced clinical symptoms, i.e., an influenza-like syndrome and, in three cases, the classic *erythema migrans* associated with Lyme borreliosis. These patients were treated with antibiotics. We obtained the biting ticks from two of these symptomatic patients (one nymph and one female of *I. ricinus*). Moreover, physicians reported the prescription of antibiotics and antihistaminic drugs in two other patients bitten by ticks, though they were asymptomatic.

### 3.2. Detection of Tick-borne Pathogens in Biting Ticks

Molecular analyses disclosed an overall infection prevalence of 25.4% (*n* = 30 positives out of 118 tested ticks; 95% CI= 17.9–34.3) in feeding *I. ricinus* retrieved from human patients. Spotted-fever group Rickettsiae were the most prevalent bacteria and infected 22.0% of investigated ticks (*n* = 26; 95% CI = 14.9–30.6), namely one larva, 20 nymphs, and 5 adults. *Anaplasma phagocytophilum* infected two nymphs and one female (2.5%; 95% CI = 0.5–7.3), while Lyme spirochetes were detected in three nymphs and one female (3.4%; 95% CI = 0.9–8.5). Double infections were identified in two ticks (1.7%; 95% CI = 0.2–6.0), with SFG rickettsiae and *A. phagocytophilum* co-infecting one *I. ricinus* female, and Lyme spirochetes and SFG Rickettsiae co-infecting an *I. ricinus* nymph. 

By nucleotide sequencing, we identified two *B. burgdorferi* genospecies: *B. afzelii* and *B. garinii*. Nucleotide sequences were identical to those identified in questing *I. ricinus* from high Susa Valley (GenBank accession numbers MT038899 and MT038900) [10]. *Rickettsia helvetica* and *R. monacensis* infected 15.4% (95% CI = 4.4–34.9) and 30.8% (95% CI = 14.3–51.8) of the rickettsiae-positive *I. ricinus*, respectively. Due to the poor quality of the sequences, we failed in the identification of SFG Rickettsiae infecting 14 of the feeding ticks. *Rickettsia helvetica* sequences (*n* = 4) showed 100% of similarity to *R. helvetica* isolated in *I. ricinus* from Switzerland (U59723.1). *Rickettsia monacensis* sequences (*n* = 8) were identical to those previously identified in the study area in questing *I. ricinus* (MT025711) [10]. 

Regarding the two ticks collected on symptomatic patients, *B. afzelii* infected the nymph, and *R. monacensis* infected the female *I. ricinus*. 

*Rickettsia slovaca* was detected in the two *D. marginatus*, as previously reported [11].

### 3.3. Public Awareness about Ticks and Tick-Borne Diseases

We collected 495 completed questionnaires during 17 informative meetings. Respondents were 280 males, 208 females, and 7 people who did not provide gender-related data. The mean age was 57 years (minimum–maximum: 8–88), with pensioners being 35.7% of the sample. Most of the respondents belonged to the ‘general’ public (*n* = 344; 69.5%, 95% CI = 65.2–73.5), followed by specific at-risk categories: hikers (*n* = 86; 17.4%, 95% CI = 14.1–21.0), hunters (*n* = 49; 9.9%, 95% CI= 7.4–12.9) and mushroom pickers (*n* = 16; 3.2%, 95% CI = 1.8–5.2; Table 2). 

Two hundred participants (40.4%; 95% CI = 36.0–44.8) declared that they had suffered tick bites at least once, mainly during the summer (named by 73.3% of respondents) and spring (36%). A lower proportion of respondents reported tick bites occurring during autumn (11.6%), while two people reported tick bites occurring all year round. Tick bite events were not significantly associated with any category of respondents (Fisher exact test, *p* = 0.11), although hunters reported a higher percentage of tick bites (Table 2).

Most tick-bitten people (*n* = 154) declared that they removed the ticks autonomously, while a low proportion (*n* = 30) received health services. The first group enumerated several procedures for tick removal: the use of tweezers was the most common method reported (*n* = 52 people), followed by the use of oil or chemicals (*n* = 10), removal with fingers (*n* = 9), and by taking a shower (*n* = 2); one respondent burned the tick with a cigarette. After removal, only 13 respondents disinfected the bite area and four respondents declared the use of antibiotic therapy. 

Almost half of the people surveyed (46.2%; 95% CI = 41.8–50.7) claimed they usually adopt preventive measures to avoid tick bites. The odds of adopting preventive measures were significantly higher in people having previously experienced a tick bite (OR = 3.4; 95% CI: 2.3–5.2); gender, age, and category of respondents did not affect the likelihood of adopting protection. Protection measures were indeed implemented by 63.5% (95% CI = 56.4–70.2) of people who had experienced tick bites, compared to 34.6% (95% CI = 29.2–40.3) of people who had never been bitten. Appropriate clothing was the most common preventive measure undertaken by respondents (68.1%; 95% CI = 61.6–74.1), followed by the use of repellents (31.0%; 95% CI = 25.1–37.4), and the visual inspection of the body (12.7%; 95% CI = 8.6–17.7). Avoiding visits to risky areas or staying in footpaths during recreational outdoor activities were also mentioned by 10.0% (95% CI = 6.5–14.7) of participants. In some cases, more than one measure was adopted. Hunters were less prone to protect themselves against tick bites (Table 2), although the difference among categories was not significant. When considering the specific behaviors adopted, only the use of repellents was associated with the gender of respondents, with male interviewees having lower odds of applying them (OR = 0.5; 0.3–0.9).

Most of the people who owned domestic animals stated that they protect them from ticks (79.3%, 95% CI = 73.4–84.4). Almost all were familiar with antiparasitic treatments (95%; 95% CI = 90.7–97.7) and two people indicated they routinely perform visual inspections for ticks. 

Ticks were considered a health risk by most respondents, with 70.3% of people (95% CI = 65.5–75.0) stating that they are vectors of infections. Some respondents specified the name of some diseases: Lyme borreliosis (*n* = 28), tick-borne encephalitis (*n* = 9), and rickettsioses (*n* = 3). Almost three quarters of the participants (69.1%; 95% CI = 64.8–73.1) had previously heard about the hazard posed by ticks, in particular tick-transmitted infections. Mass media, such as TV and newspapers, were the main information sources reported for knowledge on ticks (41.2%; 95% CI = 36.0–46.6). Other information sources mentioned were their acquaintances (28.9%; 95% CI = 24.2–34.1), health professionals, such as veterinarians (12.3%; 95% CI = 9.0–16.2), physicians, and/or pharmacists (8.5%; 95% CI = 5.7–12.0), professional figures linked to nature (6.1%; 95% CI = 3.8–9.2), universities, and/or research institutes (5.2%; 95% CI = 3.1–8.1).

## 4. Discussion

Our research indicates that: i) people inhabiting or visiting natural areas of Piedmont region, northwestern Italy, are exposed to infected tick bites; ii) the generally low level of awareness and subsequent protection may potentially enhance the likelihood of contracting tick-borne diseases. In fact, although the majority of interviewees in the study area recognized ticks as a threat to their health, and around 40% claimed to have suffered from tick bites, the adoption of individual protective measures against tick bites seems insufficient. Even worse, we recorded that people generally notice and remove ticks when they are already engorged; in the case of infected ticks, delayed removal is known to substantially increase the chance of pathogen transmission. 

The use of individual protective measures is the best practice to protect oneself from tick bites [24]. Such measures include wearing protective clothing (long sleeves and trousers, tucking trousers into socks, wearing light colors), using tick repellents, avoiding infested areas, or staying on trails, checking the body for ticks to remove them promptly, and showering after visiting a risk area [25]. A higher risk perception by citizens is generally associated with an increased use of these measures [24], although this is not always observed in endemic areas [26,27]. In our study, less than half of the questionnaire’s respondents declared the adoption of preventive measures against tick bites. Protective clothing was the mostly used measure, followed by the use of repellents (around 68% and 30% of respondents who adopted measures, respectively; Table 2). Surveys performed in Finland [28] and Sweden [24] showed a greater use of protective measures among their respondents (> 60%); however, comparable results were observed regarding the type of measures adopted (60–80% for clothing vs. 16–24% for repellents). Of note, less than 5% of our respondents avoided visiting areas infested by ticks versus 61% of respondents in Finland and 43% in Sweden. It is reasonable to assume that, due to the novelty of tick presence in our study area, people are not fully aware of the characteristics of zones at greater tick risk. Moreover, hunters and mushroom pickers in our sample seem to adopt few precautions, despite they routinely abandon the trails and intensively explore risk areas during their activities. Finally, only 5.9% of our interviewees stated to self-inspect their body after outdoor activities, a habit that is instead adopted by 50–60% of the interviewees in the Scandinavian studies. Our results are in accordance with previous research [8], which reported a low adoption of preventive measures in areas of new emergence of Lyme borreliosis, compared to endemic areas.

The infrequent habit of checking the body among our interviewees is reflected in the high engorgement level of tick specimens removed from bitten people: ticks are probably noticed only when they are engorged and, thus, more visible, or more easily perceived by touch. Indeed, our estimation of the attachment duration based on the tick engorgement rate indicated that ticks were generally attached for 48–72 h, and in some cases exceeded 96 h. On occasion, the high engorgement rate was due to the long time elapsed from tick discovery, and the moment when patients went to the emergency unit and were visited by the attending physician. Available guidelines state that the risk of infection is lower with prompt and accurate tick removal. In particular, to avoid Lyme borreliosis transmission, ticks should be removed within the first 24 h after the bite [29]. Exceptions exist, with reports of early *Borrelia* spp. infections after short exposure to *Ixodes* tick bites [30,31,32] and other pathogens being efficiently transmitted within a shorter period or without time delay, e.g., some SFG Rickettsiae [33] or TBEV [34]. Nevertheless, the duration of tick attachment increases the probability of pathogen infection [35,36,37,38]. In our study, nymphs were the most prevalent life stage biting humans; their small size increases the possibility of remaining unnoticed, and leads to a longer attachment duration [17,39].

Regarding tick removal, most people used tweezers, but we also recorded the adoption of inappropriate procedures, such as tick crushing, the attempt to burn attached ticks with cigarettes or the application of oil or chemical substances before tick extraction. The application of chemicals on attached ticks was proved ineffective, and inadequate tick management during removal may increase the risk of pathogen infection [40]. Few people declared to disinfect the bitten area after tick removal—a practice explicitly suggested by the European Centre for Disease Prevention and Control [41]. 

Previous studies showed that the sociodemographic characteristics of respondents might influence the adoption of preventive measures. For example, in endemic regions of Sweden [24] and Switzerland [8], women were more likely to adopt protective measures against ticks, while young people seemed less willing to wear protective clothing. Our results suggested a more frequent use of repellents by women, but no other differences were found between gender and among age classes. In line with this, we did not detect differences in the adoption of individual protection measures between the ‘general’ public and the categories at risk (hikers, mushroom pickers, hunters). 

We acknowledge limits in our questionnaire study. First, some ‘at risk’ categories were scarcely represented in the respondents’ sample. Second, the sample was not randomly chosen: we may hypothesize that people attending our informative meeting were generally interested in the subject. However, this may imply that they were also more sensitive to the health hazard posed by ticks, and perhaps more prone to the adoption of protective measure, so our results seem to indicate a low general knowledge of ticks and the related preventive measures in the study area. Moreover, less than half of respondents adopted protective measures, even among the most exposed categories (Table 2); this suggests that an increased exposure does not necessarily result in greater awareness. 

Interviewed people who had previously suffered from tick bites were more prone to adopt precautionary measures, which may be dictated by the information gained on the subject after the bite event. This is in accordance with previous research [27], which reported greater awareness in people already tick-bitten or having suffered from a tick-borne disease.

Considering that ticks represent a growing health concern, it might be reasonable to expect health professionals, such as medical doctors, veterinarians, or pharmacists, to be identified as the main source of information for citizens. However, based on our survey, most information on ticks and related health risks is acquired through the media. This was also observed in Finland [28]. Since, in our context, tick-borne diseases are relatively rare events, such information is likely still not prioritized by general physicians. Interestingly enough, our interviewees seemed to pay more attention to protecting their pets against ticks, with almost 80% of respondents claiming to regularly apply antiparasitic drugs. This suggests that pets are prophylactically treated within a broad perspective of ectoparasite control, without special attention to threats specifically posed by ticks.

Although tick sampling in this study was based on the possibility/willingness of general physicians to collect ticks on patients and the voluntary provision of these ticks, we observed patterns in the occurrence of tick bites similar to other studies. Previous research showed a higher proportion of tick bites in children, often localized in upper regions of the body [17,42,43]. We also found that children (<10 years old) are particularly vulnerable, accounting for approximately 26% of bite cases. This might be due to the greater attention by parents to the hygiene of their children, which helps in detecting feeding ticks on the body. Our data identified *Ixodes ricinus* as the tick species most frequently involved in bites, with nymphs being by far the prevalent stage. Other studies have recently reported similar results in the Piedmont region, indicating the wide distribution and abundance of *I. ricinus* [44,45]. Approximately 80% of the bites were recorded during spring–early summer, corresponding to the peak of *I. ricinus* in the study area [10]. *Dermacentor marginatus* seemed to play a secondary role in tick bite events, in line with previous studies carried out in Italy [46], where this species was deemed responsible for only 4.5% of recorded bites. In the study area, we registered low numbers and a focal distribution of questing *D. marginatus* [11]. 

We detected several zoonotic agents in ticks collected on human patients. *Rickettsia* spp. were the most prevalent, including *R. helvetica* and *R. monacensis* in *I. ricinus*, and *R. slovaca* in *D. marginatus*. *Rickettsia slovaca* is one of the main causative agents of tick-borne lymphadenopathy (SENLAT syndrome) [47], while the pathogenicity of *R. helvetica* and *R. monacensis* is still poorly understood [48,49]. *Rickettsia* spp. were detected in *I. ricinus* larvae, which is explained by the efficient transovarial transmission route described for these tick-borne bacteria [50]. *Anaplasma phagocytophilum* is the causing agent of human granulocytic Anaplasmosis (HGA), which commonly presents non-specific symptoms in humans [51]. Unfortunately, we could not identify the *A. phagocytophilum* ecotype involved in tick infections, but we have recently detected the zoonotic ecotype I in questing *I. ricinus* from the same study areas [10]. By contrast, *Borrelia burgdorferi* s.l. is one of the most common transmitted bacteria along the distribution range of *I. ricinus*. We detected a relatively low proportion of Lyme-infected ticks, compared to the 15.5% of prevalence in questing ticks from the same study area [10]. However, we tested a limited sample of feeding ticks in this study; moreover, previous surveys on ticks feeding on humans in the Piedmont region showed contrasting results, with prevalence ranging from 0% to 11.5% [44,45]. Although several Lyme spirochetes have been detected in questing nymphs in the area [10], we only identified *Borrelia afzelii* and *B. garinii* in ticks from human patients. Both genospecies were previously associated with specific clinical presentations of variable severity, with *erythema migrans* being the most often reported [52]. 

Only 5 out of 39 tick bites reported by collaborating physicians in this study were associated with overt clinical symptoms. Infection by pathogens was identified in ticks from two of these patients, but also in ticks attached to patients that were asymptomatic when the tick was removed. The fact that a patient showing *erythema migrans* had a biting tick positive for *R. monacensis* might possibly imply he had another tick bite, from a tick positive for *B. burgdorferi*, causing the clinical status. Although tick positivity does not have any diagnostic value, it may serve as guidance for health professionals to address laboratory diagnostics during the follow-up of their patients and therapy prescriptions. 

Several factors limited the information we could have gathered from this study. In particular, it was not possible to collect ticks from all bitten patients; data accompanying the tick bite events were generally not complete; we had no access to information on the follow-up of bitten patients. Moreover, it was not possible to retrieve data on tick bite events in the previous years from the hospital electronic patient record system, to highlight any possible fluctuation in their incidence. A greater involvement of general physicians in data collection could enable a more representative picture of the sanitary impact of ticks in the study area. In the Netherlands, for instance, general practitioners routinely report tick bites and disease cases, even though tick-borne diseases are not notifiable diseases. Such reports have enabled the identification of risk areas for Lyme borreliosis throughout the country [53], and informed health authorities about the incidence of tick bites and Lyme borreliosis [54,55], while assessing their impact in the public health [38,56].

## 5. Conclusions

In our study area, poor awareness of the hazard posed by ticks and tick-borne diseases may result in an increased risk of exposure to infected tick bites. Understanding how people perceive the risk can be useful to policymakers and health authorities, to communicate with the public and direct educational efforts [57,58]. Such efforts should aim to increase the knowledge on arthropod vectors and promote the adoption of preventive measures and habits [7,59]. In particular, categories at-risk for occupational or leisure reasons should be targeted. Forest rangers, for example, are among these, and their professional statures may be helpful in increasing tick awareness, while still participating in outdoor recreational activities. Accordingly, medical doctors could be key in providing updated and evidence-based information to people. Finally, greater collaboration among health professionals should be encouraged to obtain reliable data on the incidence of bite events and tick-borne diseases. 

## Figures and Tables

**Table 1:** Estimated attachment duration (in hours) of feeding *I. ricinus* nymphs and females removed from humans; Turin province, 2017–2020.

Hours of Attachment	Nymphs		Females	
	*n*	% (95% CI)	*n*	% (95% CI)
0–24	0	-	0	-
24–48	9	12.7 (6.0–22.7)	0	-
48–72	61	85.9 (75.6–93.0)	6	54.5 (23.4–83.2)
72–96	1	1.4 (0.04–7.6)	2	18.2 (2.3–51.8)
>96	0	-	3	27.3 (6.0–61.0)

**Table 2:** Percentage of respondents to the questionnaire reporting tick bites and the adoption of protective measures against ticks, by category of respondents; Turin province, 2017–2019.

Questionnaire Item	Respondents’ Category (%, 95% CI)				
	General Public (*n* = 344)	Hikers (*n* = 86)	Hunters (*n* = 54)	Mushroom Pickers (*n* = 16)	Total
Bitten by ticks	39.2 (34.1–44.6)	39.5 (29.1–50.7)	50.0 (36.1–63.9)	33.3 (9.9–65.1)	40.4 (36.0–44.9)
Adopts prevention measures	47.7 (42.3–53.1)	44.2 (33.5–55.3)	37.0 (24.3–51.3)	43.8 (19.8–70.1)	46.3 (41.8–50.7)
Wears protective clothing	31.4 (26.5–36.6)	31.4 (21.8–42.3)	31.5 (19.5–45.6)	25.0 (7.3–5.4)	31.5 (27.4–35.8)
Applies tick repellents	14.5 (11.0–18.7)	13.9 (7.4–23.1)	7.4 (2.1–17.9)	37.5 (15.2–64.6)	14.5 (1.2–18.0)
Checks body	6.4 (4.1–9.5)	4.6 (1.3–11.5)	5.6 (1.2–15.4)	0	5.9 (4.0–8.3)
Avoids risk areas	5.2 (3.1–8.2)	5.8 (1.9–13.0)	0	0	4.6 (2.9–6.9)
Adopts more than one prevention measure	46.5 (38.6–54.6)	11.5 (5.4–20.8)	8.5 (2.4–20.4)	18.7 (4.0–45.6)	30.0 (24.9–35.5)

## Data Availability

Data presented in this study are available on request from the corresponding author, with the permission of physicians and Ente di Gestione dei Parchi delle Alpi Cozie and Po Torinese.

## Supplementary Materials

The following are available online at https://www.mdpi.com/article/10.3390/vetsci8070131/s1, Figure S1: Questionnaire administered to the public during informative meetings on ticks and tick-borne diseases; Turin province, 2017–2019.
