# Supplementary material for: Low Risk Perception about Ticks and Tick-Borne Diseases in an Area Recently Invaded by Ticks in Northwestern Italy
Source: Vet Sci. 2021 Jul 13;8(7):131. doi: 10.3390/vetsci8070131 (PMC8310202; doi:10.3390/vetsci8070131)
Supplement: Supplementary file 1 [file vetsci-08-00131-s001.zip › vetsci-1276998-supplementary.pdf]

Do you know ticks?

Age: ______ Sex: ☐ M ☐ F
Employment: ________________________________
Hometown / city: ____________________________

1) Have you ever been bitten by tick? ☐ Yes ☐ No
   If *'Yes'*,
   - Where and when did the tick bite occur?
     ____
   - What did you do?
     ____

2) On your opinion, do ticks endanger the health of humans and animals?
   ☐ Yes ☐ No
   If *'Yes'*, what kind of danger do they pose?
   ____
   ____

3) Have you heard about potential risks posed by ticks? ☐ Yes ☐ No
   If *'Yes'*, from whom? What kind of risks did they mention?
   ____
   ____

4) Do you adopt any protective measure to protect yourself from tick bites?
   ☐ Yes ☐ No
   If 'Yes', could you please specify how do you protect from ticks?
   ____

5) Do you protect your pets/animals from ticks?
   ☐ Yes ☐ No ☐ I don't own pets
   If 'Yes', could you please specify how?
   ____

Figure S1. Questionnaire (translated from Italian) administered to the public during informative meetings on ticks and tick-borne diseases; Turin province, 2017-2019.
